# The nurse in child healthcare as a craftsman to use professional competence and build a health promotive relationship- an observation study

**DOI:** 10.1080/17482631.2025.2527146

**Published:** 2025-07-02

**Authors:** Veronica Bohlin, Hrafnhildur Gunnarsdottir, Katarina Patriksson, Elisabeth Dahlborg

**Affiliations:** a Department of Health Sciences, University West, Trollhättan, Sweden; b Department of Pediatrics/Neonatology, NU-Hospital Group, Trollhättan, Sweden

**Keywords:** Collaborative partnership, community of practice, health promotion in child healthcare, interaction, participant observations, professional competence, salutogenesis, work-integrated learning

## Abstract

**Purpose:**

To explore how child healthcare (CHC) nurses use their professional competence to promote health in families with children.

**Method:**

An explorative qualitative study was conducted based on data from participant observations and informal conversations. The sample comprised 12 CHC nurses who were observed for 31.5 hours during 23 health visits, 17 phone calls, and 9 team visits, totalling 49 observations. The results were analysed using a qualitative content analysis.

**Results:**

The analysis resulted in one theme: Health promotion as a professional competence is a craft of interaction for caring and mutual learning. The theme describes how CHC nurses use professional competence as a health promotion approach to create trust, where the decision-making and guidance of parents are skills that depend on interaction, which is an ongoing process. The shared learning outcome of the encounter was dependent on a healthy learning process.

**Conclusion:**

A health promotion approach was a part of CHC nurses’ professional competence, which involves interaction for caring and sharing mutual learning. The ongoing process of healthy learning occurs through interactions between nurses, children, and families. This process can be considered as a pedagogical work-integrated learning tool.

## Introduction

Swedish child health care (CHC) support refers to the unique needs of every child and family, where the nurse´s competence is a prerequisite for identifying factors that promote a child´s healthy development (Ekström-Bergström et al., 2022; Langeland et al., 2022; Shorey, 2021). To identify these factors, a relationship with the family is desirable, where both parties are seen as collaborative partners (Benzein et al., 2008). Previous research highlights the importance of establishing mutual and trustful relationships between the nurse and the family. Such relationships serve as a bridge, enabling encounters that provide individualized support tailored to the family’s needs (Bohlin et al., 2022; Eklund et al., 2022). Health promotion interventions are not only dependent on the competence of nurses, but also on how evidence-based knowledge is implemented in the workplace (Langeland et al., 2022). Nursing is a profession in which a combination of different types of knowledge is needed such as generative, transformative and experience-based knowledge. Experience-based knowledge is developed in an ongoing process, created through experience and reflective observations (Benner, 1984; Kolb & Kolb, 2017; Schön, 1992). Specialized nurses in CHC use their experiences as part of their competence at their expert level for health promotion and it is important to understand how they use this competence in their work.

## Background

The transition into parenthood can give rise to stress for both parents and children (Gilmer et al., 2016) and being able to respond to the child´s early signals through responsive interaction is fundamental for the child´s development (Bowlby, 1956). Therefore, supporting parents with focus on their ability and empowerment to enable their participation as well as the participation of their children (RHB, 2023; Swedish National Board of Health and Welfare, 2014). Strengthening and empowering parents aligns with the World Health Organization which explains health promotion as a process of making it possible for people to increase control over and improve their health according to the 1986 Ottawa Charter (WHO, 2008).

Swedish CHC is publicly funded, free of charge and voluntary, reaching almost every child from birth up to the age of six years; hence, it is an important arena for promoting the health of young children and their families (Wallby & Hjern, 2011; Wettergren et al., 2016).

The Swedish act on United Nation Convention on the Rights of the child (SFS, 2018, p. 1197) should serve as a guide for all Swedish CHC. Nurses in CHC are specialized in paediatric nursing or public health and the teams at a CHC centre consist of nurses and physicians, where the nurses organize and lead the work as healthcare providers for the child and family. The child and family are referred to experts such as psychologists and nutritional specialists when necessary (Swedish National Board of Health and Welfare, 2014). Each area of a county or region has a Central Child Health Service Unit that is responsible for the quality of the CHC and the managers of the CHC centres are responsible for the tasks of CHC (RHB, 2023; Swedish National Board of Health and Welfare, 2014). The specialized nurse must possess comprehensive knowledge of child development, including the emotional, physical, and mental needs that children may have. Additionally, the nurse should be well-versed in strategies and interventions aimed at promoting children’s health and preventing ill health (RHB, 2023; Swedish National Board of Health and Welfare).

The nurse’s role in CHC focuses on fostering the necessary conditions for effective health promotion, while actively working to reduce social inequalities in health (Sandberg, 2022). The expanded home visiting programmes are examples of proportional universalism in targeted areas of vulnerability in Sweden (Marttila et al., 2017) as part of a more equal CHC system/service. Adopting a health promotion focus involves supporting each individual’s unique prerequisites, while also shaping environmental conditions that influence health outcomes (Lundström et al., 2020).

As parents are expected to be involved in the health promotion approach (RHB, 2023; Swedish National Board of Health and Welfare, 2014), a more collaborative partnership between nurses and parents is required, in which the parent is invited into the care and the nurse is invited to the family (Benzein et al., 2008, 2023).

Nurses’ competence is both practical and theoretical. To handle theory and practice as equally valid components of nursing competence, it is important to assign the same level of relevance to both generative and transformative forms of knowledge (Oberle & Allen, 2001).

In this study, a workplace in a CHC setting is seen as a community of practice formed by people engaged in a process of collective learning. Nurses in CHC have regular interaction with each other which teaches them how to share problems so that collective learning emerges.

Vygotskij (1978) described the central idea that learning happens when human activities take place in a social and cultural context, where shared social activities are transformed into new knowledge that continues to interact with and be transformed within a reciprocal relationship within the internal process (Vygotskij, 1978).

A salutogenic umbrella framework can serve as a fertile environment for optimal health and well-being during the first period of a child´s life (Shorey, 2021). Hence, for a balanced approach to health, it is important that both salutogenic and pathogenic approaches complement each other (Antonovsky, 2005; Langeland et al., 2022; Lindström & Eriksson, 2005).

Both parents’ commitment and participation are important for children’s development, and CHC nurses need to be aware of their own supportive abilities. Organizing supportive activities enables parents to perceive their own unique needs (Hrybanova et al., 2019) and since the importance of sufficient time to support parenthood in clinical practice has been highlighted in previous research, lack of time was perceived as a major barrier in daily work by CHC nurses (Ersson et al., 2021).

Salutogenesis presents an individual’s resources such as determinants that enable people to develop a sense of coherence (Antonovsky, 1979). According to Nilsson and Lindström (1998), both the Ottawa Charter and the determinants can be seen as learning processes in which people interact with each other and learn through experiences. The ability to learn will develop health, a sense of coherence (Nilsson & Lindström, 1998) and also promotes health by describing learning as a process of healthy learning (Lindström & Eriksson, 2011). The process of relating to others creates learning, and the knowledge gained from practice expands the space for knowledge (Pennbrant & Svensson, 2018).

Ongoing learning throughout work life has become more important for many professions because of occupational challenges and requirements. Rapid changes in society and working life require learning in the workplace as a prerequisite for lifelong learning (Tynjälä, 2008). For lifelong learning in the workplace, it is important that professionals have opportunities for learning at work, which can be seen as a manifestation of work-integrated learning (WIL) (Billett & Choy, 2011; Björck & Willermark, 2024). WIL includes work-based learning experiences that can lead to the development of different types of knowledge and enable learning from both theory and practice (Björck & Willermark, 2024).

Nurses in Swedish CHC are asked to contribute evidence-based practice based on experience and knowledge of the national child health service programme. One of the tasks is to use health promotion and identify the unique needs of each child and family to promote physical health and support a healthy lifestyle (RHB, 2023; Sandberg, 2022; Swedish National Board of Health and Welfare, 2014).

The task for specialized CHC nurses is to promote the health and well-being of the child and family, for which a collaborative partnership between nurses and parents is required. Studies of CHC show that competence is a prerequisite when promoting health and identifying factors to support the child and family. However, there is a gap in the body of knowledge on how nurses use their competence and professional judgement in their health promotion work. Observing how health promotion emerges in the encounter between the nurse and family makes it possible to highlight this.

This study aimed to explore how CHC nurses use their professional competence to promote health in families with children.

## Materials and methods

### Design

The study had an exploratory qualitative design, using participant observations and informal conversations (Polit & Beck, 2021) with nurses at their workplace in CHC centres. The participant observations were inspired by the ethnographic research approach, the process of learning about people by learning from them (Roper & Shapira, 2000). In participant observations, the observer engages with a group for a period in a natural setting in order to develop an understanding of the group (Foster et al., 2021).

### Study setting

The five CHC centres were in areas with different contexts to obtain as broad a selection as possible for purposive sampling and to ensure variation. Inclusion criteria for CHC centres were being located in either vulnerable or well-functioning/affluent socio-economic areas, suburban or urban areas, as well as smaller and larger workplaces, with two to six nurses employed at each CHCC. In Sweden, all CHC nurses are registered nurses specializing in public health or paediatric nursing. Their work, which is covered by the national child health service/CHS programme, involves regular growth and development check-ups, health counselling and follow-ups, regular vaccinations in addition to home visits for newborns, and at 8 months of age. They also participate in teamwork with physicians and collaborate with different professionals when necessary. All nurses had a set time for regular phone calls to answer the parents’ questions.

### Data collection and sample

This study was conducted from October 2022 to February 2023 at five different CHC centres in western Sweden. Eight CHC centres were invited to participate in the study, but three could not be reached or did not respond. Five CHC centres managers agreed to participate in the study and gave the researchers permission to invite nurses to participate. The chosen CHC centres are within primary care; all of them are publicly financed and are either publicly or privately managed.

Fourteen nurses were invited by the first author via phone or email, and twelve of them agreed to participate in the observational study.

All of the nurses signed informed consent form before participating (see Table I). They all worked as CHC nurses, four specialized in paediatric nursing, and eight in public health nursing. Their mean experience of work in CHC was 8.17 years and their ages ranged from 36 to 60 years (mean, 49 years). Table I includes the participants. To maintain anonymity, all were de-identified with fictitious names.

**Table I.** Description of the participants.

Participant	Specialisation	Years of experience
Alice	Public health	11
Brita	Public health	12
Cornelia	Public health	6 months
Dagny	Public health	20
Ebba	Public health	13
Fredrika	Public health	18
Gudrun	Paediatric Nurse	5 months
Hildur	Paediatric Nurse	2
Ida	Public health	3½
Jessica	Paediatric Nurse	4
Kajsa	Public health	4½
Lovis	Paediatric Nurse	10

All families gave their consent to have an observer present during their health care visit and each observation involved new children and families.

They all received oral information from both the nurse and the observer (the first author) and were also offered written information about the study stating that they could withdraw at any time that the result will be reported for academic purposes and be published in a scientific journal. They were also informed that no single individual could be identified.

The observations took place in the nurse’s natural setting, a room for health visits, team visits and phone calls. The phone calls were at a set time and on speaker. During the phone calls, only the nurse’s reactions were observed, as it was not possible to observe any interactions.

During the participant observations, the first author (VB) as the observer, sat on a chair behind the visitors, often in a corner, so that the visitors would not be confused and would be as natural as possible. During the phone calls, the first author (VB) sat next to the nurse. The observations started with the first author (VB) or the participating nurse asking the families for permission to observe the encounters. The observations consisted of 23 health visits, 17 phone calls, and nine team visits by a nurse with a physician (see Table II). A total of 49 observations were conducted, lasting 31.5 hours. Health visits at the CHC centre involved observations with nurses and visiting families with children. Nurses and children and families were present during the observations. Informal conversations after each observation were used when the observer needed answers from the participating nurse about an observed situation.

**Table II.** Type of observation.

Type of observation	Number of observations	Hours of observation (h)
Health visit Nurse	23	20
Phone calls Nurse	17	4.5
Team visits of nurse with physician	9	7

It could clarify a situation that was not obvious from the field notes on each observation (Polit & Beck, 2021). According to Foster et al. (2021), handwritten field notes can be of assistance and during the observations such notes were made at quite a high speed, while attempting to keep them clear and detailed. The field notes included descriptions of the interactions between the nurse, child and parent. The handwritten notes were reviewed for clarity shortly after each observation. The observation lasted for the entire health visit, team visit, or phone call period.

### Data analysis

To identify how nurses use knowledge to promote health, an inductive qualitative content analysis was conducted (Graneheim & Lundman, 2004; U. Graneheim et al., 2017). The analysis aimed to identify patterns, differences, and similarities, and was performed in several steps. In the first step the first author (VB) read the text to become familiar with it and to gain an initial understanding. The second step involved dividing the text into meaning units related to the aim and context. In the third step codes were created from the meaning units that retained the manifest content. The codes were then discussed with the other authors and sorted into subcategories and categories (see Table III). The work of abstracting codes and subcategories by describing similarities and differences led to categories corresponding to the aim. Categories were abstracted and interpreted based on their latent content (Graneheim & Lundman, 2004). Thereafter, a theme emerged.

**Table III.** Example of coding process.

Meaning units	Condensed meaning units	Code	Subcategory	Category
*“The child should receive treatment with laxative for as long as she needs it”* *(Alice)*	The child should receive treatment for as long as she needs it	Information about treatment	Sharing to learn	Decision-making
*“When you come straight from preschool, you should be dirty up to your ears, that´s how it should be”* *(Cornelia)*	Coming straight from preschool, the child should be dirty up to the ears	Pointing out and normalizing	Normalizing concerns	Guiding parents

### Ethical considerations

Ethical approval was obtained from the Swedish Ethical Review Authority, registration number 2022–03858–01. The study was conducted in accordance with the ethical research guidelines outlined in the Declaration of Helsinki (WMA, 2024). Participants received written and verbal information about the purpose of the study and gave written consent. All data were handled confidentially and transcribed audio-recorded materials were coded and pseudonymized. Collected data will be saved and stored in a fire-proof cabinet for 10 years, as well as on a safe server in accordance with University West data storage guidelines.

## Results

### Description of the environment/study setting

The waiting room was the first impression of the family. Some children’s furniture was found in all the CHC centres and nurses´ rooms, for the children to use. Immediately after entering the room, almost all the children who could walk went directly to the miniature furniture. There are different ways to decorate the family waiting room. Pictures of children playing were common, as well as information about various meetings, training courses on infant massage, child cardiopulmonary resuscitation (CPR), and parenting groups. There was often a nursing chair and toys for children of different ages, big toys such as tents, and small ones such as rattles.

At the CHC centre, all nurses’ workplaces were equipped with a desk, a computer, an office chair for the nurse, and one or two chairs for visitors. There was also equipment for the nurses’ work, such as a changing table with measuring equipment for small children and a baby scale, and measuring equipment for older children, including a scale to stand on. Every nurse’s visiting room contained children´s furniture, a table for drawing, and various toys for children of different ages. The equipment for the assessment of the children, such as the bags for the visits of the 2½ years old, was always out of the children´s sight, locked away, or on a high shelf.

How nurses promoted health in families with children resulted in one overarching theme: health promotion as a professional competence is a craft of interaction for caring and mutual learning.

The health promotion approach in nurses’ encounters with families appears in the interactions between the nurse and the parent and child. It comprises nurses’ way of caring for parents and children, speaking to them and actively listening to their concerns. Furthermore, it comprises the nurses’ way of acting on the parents’ concerns and their way of using the parents’ unique knowledge about their child to guide parents in their parenthood. Thus, the professional competence of the health promotion approach can be seen as a skill involving interaction and mutual learning. This skill can be summarized in three categories where nurses used their competence to create trust and support, make decisions and guide parents.

The themes and the three subcategories are presented in Table IV.

**Table IV.** Overview of categories and subcategories.

Overarching theme	Health promotion as a professional competence is a craft of interaction for caring and mutual learning		
Category	Creating trust and support	Decision-making	Guiding parents
Subcategory	Confirming parents	Reaching the child for interaction and cooperation	Normalizing concerns
	Dealing with barriers	Sharing in order to learn	Setting boundaries
		Not having all the answers	Follow- up

### Creating trust and support

Creating trust and a supportive relationship in the encounter involved confirming parents and building trust as well as dealing with barriers.

#### Confirming parents

The nurses confirmed parents by genuinely paying attention to them in the encounter and treating them with respect. How the nurses paid attention to the parents to reach the family was already observed in the welcome in the waiting room. In this way, the nurse obtained information from them before the examination. What seemed particularly important for the nurses was to start the welcome by looking at the parents during the greeting as a way of confirming and her attention was focused on the well-being of the family.

A family that had recently moved to the area came for a regular visited the CHC centre and nurse Dagny, with their firstborn, 6-week-old child. The observations showed how Dagny created trust by getting to know them, exhibiting interest, mutual understanding, and knowledge. The parents sat on two chairs opposite Dagny and talked about various things such as the move and what the family network was like.

The parents had questions about parenting groups and activities for babies, to which Dagny responded by telling them what age the child should be before starting an activity and suggested a parent group as they showed interest in joining one, which was confirmed by the parents asking Dagny to sign them up. After the visit they followed the advice and signed up for a parent group.

An already established trust could be demonstrated through the parent beginning to tell the nurse about the family´s well-being immediately after the welcoming phase without waiting for the nurse´s questions.

One noticeable outcome of confirming the parents was observed when the nurse engaged in praising the young child during conversations. These interactions often occurred at the changing table, either before or after the child’s assessment. As a result, communication with both the parents and the young child became more seamless and efficient.

Nurses used their practical knowledge to build trust during encounters with families. For example, sitting on the floor during the examination was at the same level as the child. In this way, they invited the child to an interaction at the child’s level and made the unique encounter relaxed and trustful.

#### Dealing with barriers

Sometimes, the nurses had difficulties building a trusting relationship due to various barriers that hampered the encounters. A barrier could be a lack of mutual listening, in which the nurse and parent failed to listen to each other. It could be about a mother not feeling well after giving birth resulting in her not being receptive to the nurse or the nurses felt stressed about being late because of their tight schedule.

Such barriers could lead to poor conditions for interaction and thus difficulties in building a trusting relationship. Because of the tight visiting schedule, lack of time risked becoming a barrier to optimal interaction.

For example, during one visit, nurse Alice missed a child´s signals due to lack of time.

A 3-year-old child came with a parent for a team visit to undergo a speech check. The child did not speak one understandable sentence during the visit. The parent spoke the entire time, and the child was very active. Alice did not pick this up further because of the tight schedule and ended the meeting quickly, instead of acting on the child´s signals about having speech problems.

In this case, the lack of time became a barrier to mutual listening, and the nurse was unable to combine her theoretical and practical knowledge to meet the needs of the child. In an informal talk with Alice after the visit, the nurse explained that a new appointment was planned for the child and family. The nurse also confirmed that stress hampered the encounter due to lack of time and struggled to keep to the schedule.

Another health visit included a mother with an 8-week-old baby, where the mother underwent routine screening for postpartum depression.

During the visit, the nurse Alice spoke to the baby, recognized a responsive smile, and confirmed it by smiling even more and giving praise. However, the mother did not listen to the nurse and stated that the baby was later smiling than the sibling, despite the fact that the child was not late smiling at all. Alice tried to explain to the mother that the attachment process might take time. The screening showed no depression, but the mother was not able to listen to Alice, and it was difficult to know if anything Alice told her was received. In an attempt to explain to the mother, Alice highlighted the importance of the attachment to the child and its development, using evidence-based knowledge while feeling unsure about whether the mother would receive and understand the information. There was no response from the mother at this point, but Alice points out that it is important for both mother and family to continue to come to the CHC centre regularly. Help was available, if needed. The visit ended with a new appointment for a return visit.

### Decision-making

Decision-making involves the nurses basing their decisions on the insights and information gathered during their interaction with the family. This included reaching the child for interaction and collaboration, mutual sharing to learn with the parents, and being aware that sometimes the nurses did not have all the answers.

#### Reaching the child for interaction and cooperation

Getting the child’s attention for an interaction began when the nurse invited the child to communicate to achieve cooperation. As the child’s participation is required during examinations to obtain reliable results, the nurse´s ability to engage with and support the child is important. It was often necessary to involve the parents to help the child participate. During the visit, the welcoming phase was often followed by asking if the child knew about the examination and had been prepared in any way. The answers from both the child and the parent guided the nurse in how to plan and proceed with the examination. When the nurse reached the child through verbal or nonverbal communication, cooperation was enabled. Due to the need to maintain the child´s full attention, the pace of assessment was fast.

The observation of a 4-year-old child showed that the child had not been prepared before the examination. Even so, the child was able to cooperate and interact with nurse Brita while sitting safely on the parent´s lap.

Another regular health visit showed a 2½ year old child cooperating nonverbally with support from the nurse through role-play. Using toys such as animals and dolls seemed important for reaching child in the interaction. When the nurses came to welcome families, they often started to role-play with the children using the toys in the waiting room. Many nurses working clinically during and after Covid have noticed that a lack of toys and child friendly equipment due to infection control hamper relationship building. In addition to supporting relationship building, CHC nurses also observe child´s behaviour and see developmental flags with toys as tools.

Nurse Cornelia asked the child about their teeth and whether they could roar like a tiger. The child opened her mouth and roared. Cornelia praised the child and continued the assessment by asking if the teeth were brushed and in response the child nodded. The child was also fully attentive when asked about the doll and if it was time to weigh it? The child nodded again and Cornelia placed the doll on the scales. They looked at the weight together, and then Cornelia asked if it was time to weigh the child and the child moved closer.

This example shows how Cornelia reached the child by communicating through play at a fast pace to maintain attention and achieve a result. The child´s cooperation was demonstrated through a playful response, even though the communication was nonverbal. Cornelia’s ability to be flexible in reaching the child is important for achieving the goals of the assessment. The nurses’ self-awareness of their competence was used to find the required tools and knowledge about how to deal with the child full of emotions through imagination and play. The observations demonstrated that other nurses achieved the result in a similar manner in different situations, showing presence while listening to the child´s needs. Another way of showing openness and awareness when a child visited the nurse at a regular 2½ year old health visit was to use a bag with various toys for developmental assessment.

Nurse Ida sat on the floor and tapped it to reach the child. Skills and imagination enabled Ida to use experience to obtain a result from the assessment by receiving attention and interaction from the child.

To understand the well-being of the babies, the nurses needed to reach and interact with them in a different way than with older children.

Nurse Gudrun spoke to a baby and received a response in the form of a smile and baby talk, and the mood rubbed off on the parents hence they also began to speak to the baby using their voice in the same positive way, praising the child with words similar to Gudrun´s.

This was a positive side effect that stimulated empowered parenting. It is important for nurses to use their individual skills to interact with and make professional assessments of the resources needed. Using the voice in a positive, encouraging way, and praising the child through eye contact was one way to reach them.

### Sharing in order to learn

Nurses and parents share to learn about the importance of knowledge exchange. Nurses possess professional knowledge of children’s health and development, while parents have knowledge of the unique child and family situation.

The observations showed that a prerequisite of sharing was to treat the parent as a valuable person to be listened to for mutual understanding. Listening to each other, and the sharing of knowledge enables learning.

The following example shows sharing to learn in a collaborative relationship between nurse Lovis and a parent during a health visit to the CHC centre, where the 12-month-old child needed an extra weight check. The family had several children and there was an established relationship.

The child was sitting on Lovis’ lap on the bench in the nurse´s reception room, and Lovis gave the child an easy puzzle representing fruits and had a conversation with the mother at the same time. They discussed words for different fruits in the native language. The parent appeared relaxed, which confirmed the nurse’s interest in and respect for the parent’s valuable knowledge. When the nurse asked the mother what the word for pear was in the native language, the parent responded with a laugh saying the word, which the nurse repeated. Still talking to each other, the nurse placed the child on the scales and recorded their weight.

In this way, the nurse demonstrated that, when using one´s competence to make an assessment, expert knowledge also consists of managing and changing roles. The parent was an expert in their native language and the nurse learned from this expertise in a collaborative relationship, in addition to words in the native language. Another example of sharing mutual understanding was when the nurse asked the parent to answer questions about the child´s development when the child refused to participate. The following example shows how nurse Alice relied on the parents´ parenting abilities and shared mutual understanding.

During a team visit, nurse Alice seemed satisfied with only the parents’ responses to follow-up on the child’s speech development. The child refused to cooperate, but the nurse demonstrated that the parents were valuable in a trusting, collaborative relationship when they shared a mutual understanding that the child would not interact during the visit.

During a team visit nurse Alice seemed satisfied with only the parents´ responses to follow-up the child´s speech development. The child refused to cooperate but the nurse demonstrated that the parents were valuable in a trusting, collaborative relationship since they shared a mutual understanding that the child would not interact during the visit.

The exchange of knowledge at the CHC centre started in a similar way at all visits and also with phone calls, in that the nurse asked questions after the greeting. If the nurse already had an established relationship with the parent, getting to the core of the matter seemed quicker, and depending on the responses, decisions were made and different actions taken. Knowledge exchange was needed to obtain valuable information about the child in order to make a professional judgement of the assessment.

An observation from a health visit where a 10-month-old child had symptoms of dry skin provides an example of how knowledge is shared as the nurse (Ida) first listens to the parent and then makes her own assessment.

The parent stood at the changing table, putting a nappy on the child after it had been weighed and said that the skin on the child’s legs felt dry. Ida touched the skin of the child´s legs and confirmed that it felt that there were dry patches. The nurse gave advice to the parents as direct information containing factual knowledge about not bathing too often because the skin was sensitive and also the importance of using a special cream for dry skin to protect it.

During the encounter, Ida listened to the parent´s concern, confirmed it, and showed respect for the parent´s knowledge by making a professional judgement. Sharing to learn equally requires a reciprocal relationship in which both parties listen to each other. Collaboration is a prerequisite for using practical and theoretical knowledge to make a professional assessment of a child´s needs.

#### Not having all the answers

The CHC nurse needed to be aware of situations where knowledge was lacking and did not always know the answer to every question. To maintain control over the situation, it is important to accept not always knowing.

Most health visits to the CHC centre were customary and routine, and parents had questions about certain symptoms or behaviour. If the nurse could not respond to the question, the parent was immediately informed. The dialogue with the parents was then usually self-explanatory, and more of a statement from the nurse that she lacked knowledge.

If the nurses realized that the necessary knowledge was lacking, they always seemed to have a plan for how to obtain it. This could include consulting a colleague at the CHC centre, psychologist or physician when required. The following example is about a 4-year-old at a regular health visit.

The parents were concerned about the child´s symptoms in different areas of tongue redness. They asked the nurse Cornelia if it was a dangerous condition. Cornelia then replied that the condition was not familiar and therefore she needed to plan further by consulting a physician to help the family.

A plan was made for further action, and the child received the support needed, as the nurse knew the importance of remaining competent, admitting her lack of knowledge, and planning further to meet the child’s needs.

### Guiding parents

Guiding parents was about creating a trusting relationship and empowering them in parenting practices. This included normalizing concerns, setting boundaries when needed, and guiding parents to care for the child´s best interests. Follow-up is required to maintain a trusting relationship. To remain competent, it was important that the nurse had a further plan for the family.

#### Normalizing concerns

Normalizing parents’ concerns was needed to prevent unnecessary anxiety over a matter that did not threaten children’s well-being. The nurse was able to convince the parents that the matter was completely normal and nothing to worry about. If the parent insisted that something was wrong, it indicated that the parent did not perceive the child´s real need. On these occasions, nurses used experience to guide parents. The following example is of a 4-year-old child, where the parent was concerned about dirt on the child´s body.

Nurse Cornelia responded that children should be dirty when coming straight from preschool, pointing out that it was normal. When telling the parent about this, the nurse used trust in the relationship as a resource, normalizing the parent´s concern. The parent seemed to listen, and there was no further mention of concern.

Normalizing a parent´s concerns in this way is an example of how nurses supported and guided parenthood based on the child´s needs. In another observation of a 10-month-old child at a health visit, the parent was concerned that no teeth would be visible in the child’s mouth.

The nurse Ebba responded that the teeth would come when they did. The parent needed further confirmation that the situation was normal, and Ebba confirmed once more that it was a completely normal situation.

The situation was similar to other concerns that needed to be normalized, thus avoiding unnecessary anxiety and supporting parents in maintaining control of the child´s real needs.

### Setting boundaries

Parents needed help from nurse to set boundaries in certain situations in the child´s best interests. The nurse guided the conversation by focusing on the child´s needs and what was good for the child. A parent phoned nurse Fredrika to get medicine and help stop the child coughing.

Fredrika tried to explain that it appeared to be a virus, and that no medicine could help. The parents seemed to push and did not listen much to what the nurse tried to explain. Fredrika had to point out in a clear voice rather abruptly that, based on evidence, there was no cure.

Occasionally during health visits, siblings joined in and sometimes took the focus away from the child to be examined. The nurse needed to devote full attention to the child and therefore had to immediately set boundaries for the sibling. Other situations were more obvious when it came to the needs of the examined children. The nurse assessed the child in silence and then clarified the outcome for parents.

This situation involved a 9- month-old child, where one of the visiting parents did not speak Swedish, and an interpreter was used. The child wore thin summer clothes, had no hats or jackets, and socks instead of shoes. Nurse Dagny first assessed the child in silence when the mother undressed the child and then checked the child´s wellbeing by talking to it, waiting for a response, and a smile. After obtaining the weight and measurements, the parents dressed the child at the changing table. Dagny explained to parents the importance of buying warm clothes for their children. The cold and rainy weather on this autumn day did not need to be used as an explanation. Nonverbal factual knowledge was evident and appeared to be the cause of the following reaction on the part of parents. The Swedish speaking parent explained the situation to the other parent in their native language. The fact that this parent also chose to say “yes” to Dagny in Swedish seemed to indicate that they both understood the seriousness of what the nurse said.

The nurse summarized the health visit, and the family was booked for a new appointment in approximately a month.

#### Follow-up

The follow-up was about maintaining the relationship with the families by summarizing and planning how to move forward, thereby maintaining and continuing the existing relationship between the nurse and the parent. Parents participated in the follow-up plan, usually through the confirmation of the next appointment. The option to decide for themselves if and when a new contact was needed showed that nurses had trust in their parenting, thus empowering them. In assessing the mother’s well-being after birth, the nurses demonstrated their professional competence and responsibility through a follow-up plan in accordance with the guidelines and the mother’s unique needs. More frequent visits for follow-up conversations with the nurse were regulated by the guidelines, which provided support to the mother. One way of measuring maternal well-being after childbirth is screening for depression and domestic violence (DVS), which is performed when the child is 6–8 weeks old.

A mother came for a health visit with 8-week-old baby. The mother filled out the questionnaires. Nurse Dagny checked the answers and explained the notice that the DVS screening showed exposure to violence “outside the family”. The mother’s response was that this is probably not unusual and that she also received help from work. Dagny pointed out that the mother has four points for well-being and then declared that the mother must be feeling well, which the mother confirmed. The nurse booked a new appointment in a month after confirmation from the mother. Dagny searched for a date that suited the unique needs of the mother for a follow-up talk within a time interval that was strategic and followed local and national guidelines.

Mothers’ confirmation of the new appointment was an important part of the follow-up, as it could make them feel that they were acknowledged and empowered by participation.

The phone calls observed also highlighted the importance of parents’ participation. They often ended with a summary of the conversation by the nurse and confirmation that a new phone call should be taken into consideration if needed, either by the nurse or the parent. By giving the parents the choice to call again if needed, trust in parenting was shown. Another way of encouraging parent participation was to ask the parents directly when they wanted to return for follow-up. Having these questions posed respectfully by the nurses conveyed a sense of participation. Nurses created opportunities for parents to build their knowledge and skills through the learning processes CHC nurses create within the nurse and parent interactions.

## Discussion

This study aimed to explore how CHC nurses use their professional competence to promote health for children and their families and our research has provided important evidence of CHC nurse practice which is lacking to date. The overarching theme: health promotion as a professional competence is a craft of interaction for caring and mutual learning, reflects CHC nurses’ work in doing health promotion. The categories encompass subcategories that illustrate the work as a craft, with interaction as a fundamental process integrated into each aspect. A recent study by Bohlin et al. (2022) highlighted that CHC nurses are bridge-builders due to their relationship with the child and family. Our results show that learning that develops from interaction is dependent on encounters between the nurse, child, and family.

As the shared learning outcome of the encounter was dependent on the interaction process, which in itself can be considered health promotion in line with the Ottawa Charter (WHO, 1986), and states that health promotion is a resource and a process to improve health. As the work of promoting health starts at the beginning of life (WHO, 2008) the CHC nurses’ work can make a difference for the children.

As a prerequisite for the ongoing process between nurses, children and families, the encounter can be considered as a movement towards health through knowledge. Furthermore, the result shows that the encounter consists of a social practice in which mutual learning occurs. Thus, the CHC centre is considered a community of practice in our study and described by Lave and Wenger (1991) as a practice of a working community since families are in specific social circumstances, the CHC centre, and are involved in a situated activity where mutual learning takes place.

The nurses’ clinical practice was to identify the child´s unique needs, as well as resources within and around the child and family. To succeed, nurses cared for the families as a unit with a child-centred focus and showed the importance of inviting them as valuable partners in the encounter, which is also the foundation of family centred care (Benzein et al., 2008). The nurses in the study invited families as valuable partners when they shared their knowledge of the child’s needs and situations. Promoting health, identifying and strengthening available resources around the child and family aligns with the salutogenic perspective of focusing on available resources around the individual (Antonovsky, 1979).

The results encompass health promotion care, showing that learning is situated in the ongoing process of relating to others, which corresponds with Pennbrant and Svensson (2018), who argue that learning gained from practice expands the scope of knowledge. Acquiring knowledge in the practice context, as in the present study, affects the learning process, which aligns with Nilsson and Lindström (1998), who described that a good learning process could enhance both the learning and development of a sense of coherence as a healthy outcome.

Nursing requires a wide range of competencies and in their clinical work, the CHC nurses needed to use both theoretical and practical knowledge, as well as time for reflection. When the nurse was unable to use her competence because of obstacles that hampered the encounter, there was a risk of ethical stress caused by missing the child´s needs and rights. Nurses could have difficulties with interactions because of barriers, such as a lack of time. This emerged when, for example, a 3-year-old child came for a speech check. Although the nurse identified shortcomings regarding language, the visit ended quickly, and the nurse confirmed that stress and striving to keep on schedule hampered the encounter, which aligns with Ersson et al. (2021), who reported that lack of time was a barrier in daily work for CHC nurses. This also hampers nurses’ opportunities to reflect both on-encounter and also after-encounter since the chance of health promotion care and a healthy outcome for the child and family is reduced because the parent is not able to participate in decisions. The risk of feelings of disempowerment increases then and also the chance of a sense of coherence will decrease due to lack of time.

Recent research by Kwame and Petrucka (2021) describes effective communication in nurse-patient interactions as a core component of nursing care and that tight time schedules and work overload are global barriers for nurses (Kwame & Petrucka, 2021). The importance of finding risk factors and identifying the child´s and family’s unique need for resources such as determinants highlights the nurses’ work in CHC as a part of health promotion that might lead to an increase in healthy outcomes and reduced risk of physical ill health.

Therefore, it seems necessary that nurses’ in CHC as safeguards of the children´s needs and rights and their development of competence based on their knowledge of their clinical practice in CHC requires time for reflection. Reflection both on-encounter and after-encounter which also aligns with Schön (1992) and Pröckl (2023) that the time for reflection on practice increases the prerequisites for developing professional judgement and as our result points out, a more healthy outcome.

Reflections on shared learning in encounters were important for nurses to respond to the unique needs of each family, and reflection-in-time was used on most occasions.

In our study, parents played a crucial role as collaborative partners, as nurses relied on them due to the young age of the children. Often, parents acted as listening partners during information-sharing and decision-making processes with the nurses. Their relatively quiet presence may be influenced by the nurses’ tight schedules observed in our study. However, a significant aspect of health promotion work involves empowering parents to utilize their resources, enabling better support for their children and families, as highlighted by the WHO (2008).

Reeder and Morris (2021) stress the importance of health professionals being proactive in fostering parental confidence, empowering them to play an active role in shared decision-making as collaborative partners. It is widely recognized that a hierarchy of power exists in healthcare, where patients depend on the nurse’s expertise, increasing the risk of disempowerment rather than empowerment (Coyne et al., 2016; Delmar, 2012; Reeder & Morris, 2021). Coyne et al. (2016) further suggest that a child-centred approach should prioritize the child’s rights, ensuring their active participation in all aspects of healthcare delivery.

Considering CHC, the observations showed that nurses are dependent on the parents during the encounter, i.e., to encourage the children to cooperate during assessments. In this way parents became collaborative partners in CHC which aligns with recent research and study of action research design that shows the importance of collaborative relationships for the nurses´ use of learning principles. A deepening learning insight for the nurses of the study as well as the learning environment for how the nurses use learning principles within parent education was also of importance (Thompson et al., 2023).

Focusing on the child while maintaining partnership with the family reflects Coyne et al. (2018) child-centred approach, which emphasizes addressing the needs of both the child and family. Health promotion for children’s rights and well-being (Swedish National Board of Health and Welfare, 2014) becomes clearer when applying the framework by Coyne et al. (2016, 2018). Their approach underscores the importance of commitment, active listening and fostering trust between nurses, children and families to provide tailored care and support.

Nurses’ diverse knowledge serves as a key resource in health promotion. Competence, as revealed in our study, involves utilizing skills in interactions to foster mutual learning and effective communication. This aligns with Fenwick’s (2006) view of competence as “performing knowledge.”

To strengthen health promotion in CHC, terms such as health-promoting care or promotion through caring might be appropriate. Ultimately, nurses’ skilled application of knowledge and interaction is vital for enhancing the process of tailored care since shared learning outcome was dependent on the healthy learning process from the interaction which is supported by research of Lindström and Eriksson (2011).

WIL could be seen as a pedagogical model to understand health promotion work and the knowledge of learning in health care (Pennbrant & Svensson, 2018). As WIL bridges both theory and practice (Sunnemark et al., 2023), it integrates and includes work-based learning experiences that can lead to the development of different types of knowledge and enable learning (Björck & Willermark, 2024).

### Strengths and limitations

A strength of this study was the use of participant observations as the methodology, which made it possible to explore how CHC nurses promote health in families with children.

The chosen method enabled a description of the nurses’ everyday work/day-to-day work in the CHC and strengthened the validity, which aligns with Larsson (2009) that participant observations form the basis for a claim of validity, as described above (Larsson, 2009).

Purposeful sampling for data collection was chosen to enable a representative selection of participants and areas, which is a strength. The high and low socio-economic, as well as urban and suburban areas in the western part of Sweden, increased the possibility of transferability to CHC and ensured credibility. The relatively small number of observation hours (31.5 h) could be considered a limitation; however, as the observations were rich in data, we do not believe that the number of hours weakens the analysis. Another possible limitation could be that not all authors took part in the data collection; however, as first author shared information about it, all authors were involved from the beginning. All authors had a preunderstanding of being nurses. To minimize the risk of bias and ensure credibility, all authors were involved in the analysis process and discussed and agreed on the results (Elo et al., 2014). To achieve dependability, all authors worked together to ensure the validity of the results and the associated categories on which they reached a consensus (Graneheim & Lundman, 2004; Lindgren et al., 2020).

## Conclusion

The results of the present study highlight the fact that the ongoing process of interaction between nurse, child and family can be considered health promotion care of a healthy outcome. A collaborative partnership with parents increases the chance of sharing decisions-making. This creates possibilities for parents to have feelings of sense of coherence, knowledge building and a strengthened empowerment in parenthood and when practical knowledge is combined with theoretical knowledge, nurses’ expertise is reflected.

For nurses’ continuous learning there is a need for a pedagogic tool to ensure experience-based knowledge in practice and to understand the important process of the health promotion care of interaction. As the ongoing process of healthy learning occurs between nurses and families, the interaction can be considered a pedagogical tool of WIL, the necessary integration of both theory and practice to understand and achieving a healthier outcome of experience-based knowledge in mutual sharing and learning.

It is important that CHC nurses value the family as a collaborative partner and, likewise, theoretically and practically, as equally valid components. When theory is not used during encounter because of a barrier, the child has increased risk of not having a healthy outcome. Drawing upon their competence in practice, there is a need for routines with set times to reflect on current situations, based on both previous experiences and theoretical knowledge.

To increase the chance of a healthy outcome and learning for the child and family it is of importance to safeguard their needs and rights. Nurses in CHC should have an awareness of being proactive in supporting parents so that they can feel empowered and participating in decision-making. Having an awareness of being proactive in supporting parents means a maintaining of the competence for the nurses and an increasing chance of a more healthy and collaborative relationship between the nurse and parent.

Future research is needed to study nurses’ perspectives on how they manage health promotion and interactions during CHC encounters.

## Data Availability

The participants of this observational study did not consent to have their documents publicly available, as they contained personal information that could be used to identify the participants.
